# Endobronchial Ultrasound Changed the World of Lung Cancer Patients: A 11-Year Institutional Experience

**DOI:** 10.1371/journal.pone.0142336

**Published:** 2015-11-06

**Authors:** Chia-Hung Chen, Wei-Chih Liao, Biing-Ru Wu, Chih-Yu Chen, Wei-Chun Chen, Te-Chun Hsia, Wen-Chien Cheng, Chih-Yen Tu, Wu-Huei Hsu

**Affiliations:** 1 Division of Pulmonary and Critical Care Medicine, Department of Internal Medicine, China Medical University Hospital, Taichung, Taiwan; 2 School of Medicine, China Medical University, Taichung, Taiwan; 3 Department of Life Science, National Chung Hsing University, Taichung, Taiwan; 4 Department of Respiratory Therapy, China Medical University, Taichung, Taiwan; 5 Graduate Institute of Clinical Medical Science, China Medical University, Taichung, Taiwan; 6 Department of Internal Medicine, Hyperbaric oxygen therapy center, China Medical University, Taichung, Taiwan; University of Utah Health Sciences Center and ARUP Laboratories, UNITED STATES

## Abstract

**Objectives:**

The role of advanced bronchoscopic diagnostic techniques in the detection and staging of lung cancer has increased sharply in recent years. The development of endobronchial ultrasound (EBUS) improved minimally invasive mediastinal staging and diagnosis of peripheral lung lesions (PLLs). We investigated the impact of using EBUS as a diagnostic method for tissue acquisition in lung cancer patients.

**Methods:**

In a single center observational retrospective study, 3712 subjects were diagnosed with lung cancer from 2003 to 2013 (EBUS was introduced in 2008). Thus, we divided the data into two periods: the conventional bronchoscopy period (2003 to 2007) and the EBUS period (2008 to 2013).

**Results:**

A total of 3712 patients were included in the analysis. Comparing the conventional bronchoscopy period with the EBUS period data, there has been a significant reduction in the use of diagnostic modalities: CT-guided biopsy (P < 0.0001) and pleural effusion cytology (P < 0.0001). The proportion of subjects diagnosed using bronchoscopy significantly increased from 39.4% in the conventional period to 47.4% in the EBUS period (P < 0.0001). In the EBUS period, there has also been a significant increase in the proportion of patients proceeding directly to diagnostic surgery (P < 0.0001). Compared to bronchoscopy, the incidence of complications was higher in those who underwent CT guide biopsy. The incidence of iatrogenic pneumothorax significantly decreased in the EBUS period.

**Conclusions:**

Advanced bronchoscopic techniques are widely used in the diagnosis of lung cancer. At our institution, the increasing use of EBUS for providing lung cancer diagnosis has led to a significant reduction in other diagnostic modalities, namely CT-guided biopsy and pleural effusion cytology. These changes in practice also led to a reduction in the incidence of complications.

## Introduction

Lung cancer is the most common cause of cancer-related death across the world[1]. It is important that patients receive a fast and accurate diagnosis in order to formulate the most adequate treatment plan. However, it remains a significant challenge for clinical physicians to diagnose lung cancer presenting as peripheral lung lesions (PLLs)[2]. The National Institute for Health and Care Excellence (NICE) guidelines for the diagnosis and treatment of lung cancer recommend choosing modalities that provide the most information about the diagnosis and staging with the least risk to the patient[3].

The two methods most commonly used to investigate PPLs and lung masses are computed tomography-guided percutaneous needle biopsy/aspiration (CT-PNB) or bronchoscopy[4]. CT-guided transthoracic needle aspiration biopsy (CT-TNAB) is an available and accepted technique for percutaneous biopsy of peripheral nodules, but it carries a significant risk of pneumothorax. Conventional bronchoscopy has a diagnostic yield of less than 20% for peripheral nodules that are less than 2 cm in diameter[5]. Therefore, it is necessary for the improved techniques to be able to sample pulmonary mass or nodules.

Advanced bronchoscopic techniques such as autofluorescence imaging (AFI)[6] or narrow-band imaging (NBI)[7], facilitate the detection of premalignant lesions and early lung cancer. Electromagnetic navigation technology (EMN) is also used for diagnosis of peripheral lesions [8]. Endobronchial ultrasound-guided transbronchial needle aspiration (EBUS-TBNA) play a key role in the diagnosis of mediastinal, paratracheal, and peribronchial lesions, as well as in lymph node staging for lung cancer[9]. Radial probe EBUS (R-EBUS) is beneficial in the diagnosis of PLLs, and together with rapid on-site cytology, R-EBUS shows high diagnostic yield [8].

EBUS is a safe and relatively accurate tool in the investigation of PPLs. It may also safely be performed under conscious intravenous sedation and is associated with very high patient satisfaction[10]. The purpose of this study was to investigate the impact of the introduction of EBUS in our institution on other diagnostic methods for tissue sampling in lung cancer patients.

## Materials and Methods

We conducted a retrospective cohort analytic study of consecutive patients diagnosed with lung cancer at the Division of Pulmonary Medicine, China Medical University Hospital—a 2146-bed community-based university hospital in Taichung, Taiwan—from 2003 to 2013. The China Medical University Hospital Internal Review Board (DMR98-IRB-335) approved the study and waived the requirements for informed consent. Radial and convex probe EBUS were introduced to clinical practice in 2008. Conventional bronchoscopy was used for lung cancer diagnosis before this period from 2003 to 2007. Data from 2003 to 2013 is included to show our change in practice over time after the introduction of EBUS. All of the data were collected, including age, sex, lung cancer pathology, subtype, diagnostic method, and complications. Selection of the modality for diagnosis was made according to the tumor position or the presence of pleural effusion. The final diagnostic method was included, for example, if bronchoscopy returned a negative result and subsequent tissue diagnosis was achieved by CT-guided lung biopsy, surgery, or pleural effusion cytology—the later method was registered. Although diagnostic modality complications such as pneumothorax, hemothorax, pulmonary hemorrhage, subcutaneous hematoma, pneumonia, acute respiratory failure, and death did occur, it was rare, fortunately.

### Statistical Analysis

The data were analyzed using SPSS, version 18.0 (Chicago, IL, USA). Continuous variables are reported as mean ± standard deviation (SD) and compared using two-tailed Student’s *t*-tests. Categorical variables are presented as the numbers of patients and percentages. Differences between categorical variables were evaluated using Fisher's exact test. All statistical tests were two-sided, and *p*<0.05 was considered significant.

## Results

Two thousand two hundred fifty-three and 4993 patients underwent bronchoscopic examinations in the conventional bronchoscopy period and the EBUS period, respectively, from 2003 to 2013. A total of 3712 subjects had a histological diagnosis of lung cancer, and other clinical data were available. In the conventional bronchoscopy period and the EBUS period, 1185 and 2527 patients, respectively had a histological diagnosis of lung cancer. The mean (SD) age was 65.58 (12.63) years for conventional bronchoscopy and 63.42 (12.68) years for EBUS (P < 0.001). The proportion of males was 66.2% (785/1185) and 61.1% (1544/2527) for the conventional bronchoscopy period and the EBUS period, respectively (P = 0.003).

### Change in diagnostic modalities used and the histologic subtypes of lung cancer

**Fig 1:**
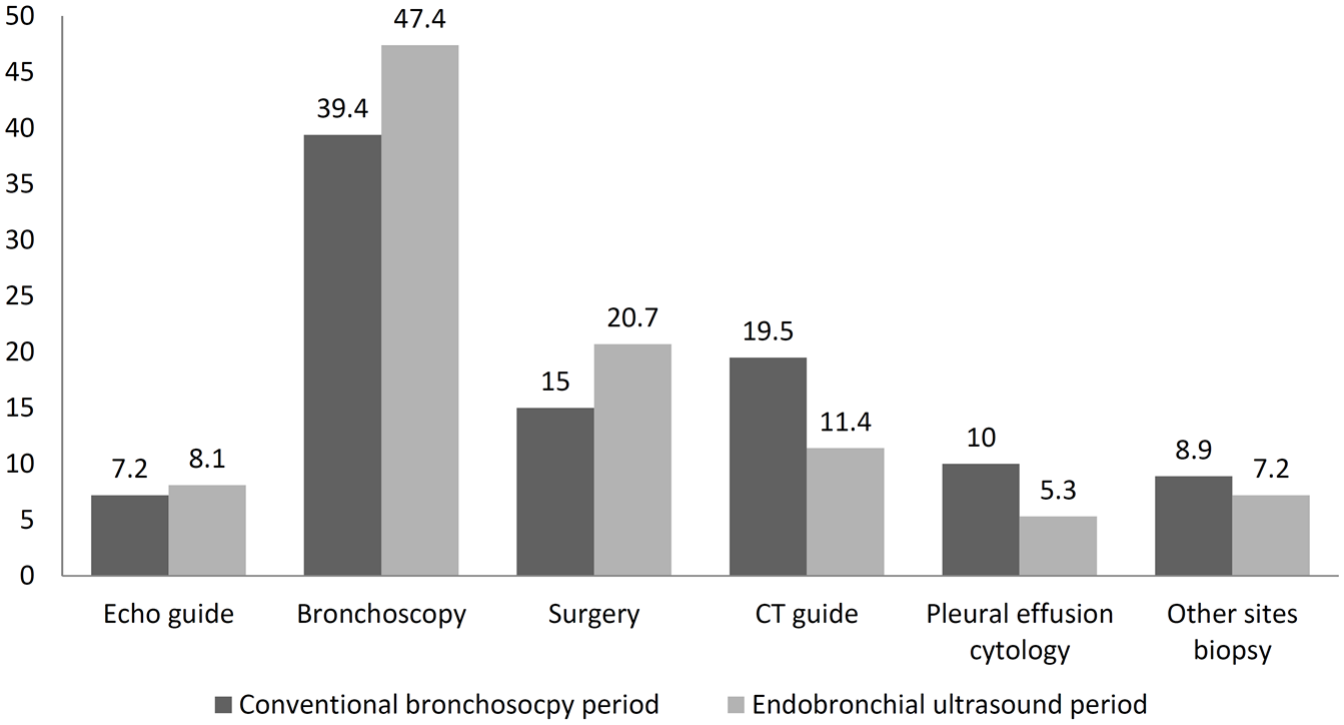
The usage of diagnostic modalities for lung malignancy between the conventional bronchoscopy and endobronchial ultrasound periods.

The histologic subtypes of lung cancer are presented in Table 1. The following are the proportion of lung malignancies between the conventional bronchoscopy period and the EBUS period, respectively: adenocarcinoma (1606/2527 [63.6%], vs. 238/1185 [20.1%], P < 0.0001), squamous cell carcinoma (598/1185 [50.5%], vs. 432/2527 [17.1%], P = 0.028), small-cell carcinoma (120/1185 [10.1%], vs. 223/2527 [8.8%], P = 0.202), large cell carcinoma (19/1185 [1.6%], vs. 70/2527 [2.8%], P = 0.029), adenocarcinoma in situ (0% vs. 41/2527 [1.6%], P < 0.0001), non-small cell lung carcinoma (59/1185 [5%] vs. 37/2527 [1.5%], P < 0.0001), and metastatic lung tumor (151/1185 [12.7%] vs. 118/2527 [4.7%], P < 0.0001). The results for the specific diagnostic method that provided the tissue for the histological diagnosis of each patient for the two periods are listed in Table 2 and Fig 1. A comparison of conventional bronchoscopy and EBUS data reveals that there has been a reduction of 8.2% (19.5% [231/1185] in conventional bronchoscopy compared with 11.3% [288/2527] in EBUS, P < 0.0001) and 4.7% (10.0% [118/1185] in conventional bronchoscopy compared with 5.3% [133/2527] in EBUS, P < 0.0001) in the proportion of histological diagnoses of lung cancer from tissue samples obtained by CT-guided biopsy and pleural effusion cytology, respectively. The proportion of subjects diagnosed by bronchoscopy significantly increased by 8.0% (P < 0.0001) between the conventional bronchoscopy (467/1185) and EBUS (1199/2527) periods. Among 1199 patients who underwent the EBUS diagnostic method, 1058 were diagnosed by R-EBUS and 141 were diagnosed by EBUS-TBNA. There has also been a significant increase in the proportion of patients proceeding directly to surgery without pathological confirmation between the conventional bronchoscopy (178/1185) and EBUS (522/2527) periods, with a 5.7% (P < 0.0001) increase in diagnoses obtained at thoracotomy. There was no significant difference in the diagnostic methods of echo-guided biopsy (P = 0.358) and other site biopsy (P = 0.065) between these two periods.

**Table 1:** The sex, age, and histological subtypes of lung cancer between the conventional bronchoscopy and endobronchial ultrasound periods.

	Total N = 3712	Conventional bronchoscopy period N = 1185	Endobronchial ultrasound period N = 2527	P value
**Sex**				
Male	2329 (62.7)	785 (66.2)	1544 (61.1)	0.003
Female	1383 (37.3)	400 (33.8)	983 (38.9)	
**Age**	64.2 ±12.7	65.6±12.6	63.4±12.7	<0.001
**Histology**				
Adenocarcinoma	2204 (59.4)	598 (50.5)	1606 (63.6)	<0.0001
Squamous cell carcinoma	670 (18.0)	238 (20.1)	432 (17.1)	0.028
Small cell lung cancer	343 (9.2)	120 (10.1)	223 (8.8)	0.202
Large cell carcinoma	89 (2.4)	19 (1.6)	70 (2.8)	0.029
Adenocarcinoma in situ	41 (1.1)	0 (0)	41 (1.6)	<0.0001
Non-small-cell lung carcinoma	96(2.6)	59 (5.0)	37 (1.5)	<0.0001
Metastatic lung tumor	269 (7.2)	151 (12.7)	118 (4.7)	<0.0001

**Table 2:** Diagnostic modalities for tissue sampling and histological diagnosis for lung malignancy between the conventional bronchoscopy and endobronchial ultrasound periods.

	Total N = 3712	Conventional bronchoscopy period N = 1185	Endobronchial ultrasound period N = 2527	P value
Echo-guided biopsy	289 (7.8)	85 (7.2)	204 (8.1)	0.358
Bronchoscopy	1666 (44.9)	467 (39.4)	1199 (47.4)	<0.0001
Surgery	700 (18.9)	178 (15.0)	522 (20.7)	<0.0001
CT-guided biopsy	519 (14.0)	231(19.5)	288 (11.3)	<0.0001
Pleural effusion cytology	251 (6.8)	118 (10.0)	133 (5.3)	<0.0001
Other sites biopsy	287 (7.7)	106 (8.9)	181 (7.2)	0.065

### Complications arising from diagnostic modalities

**Fig 2:**
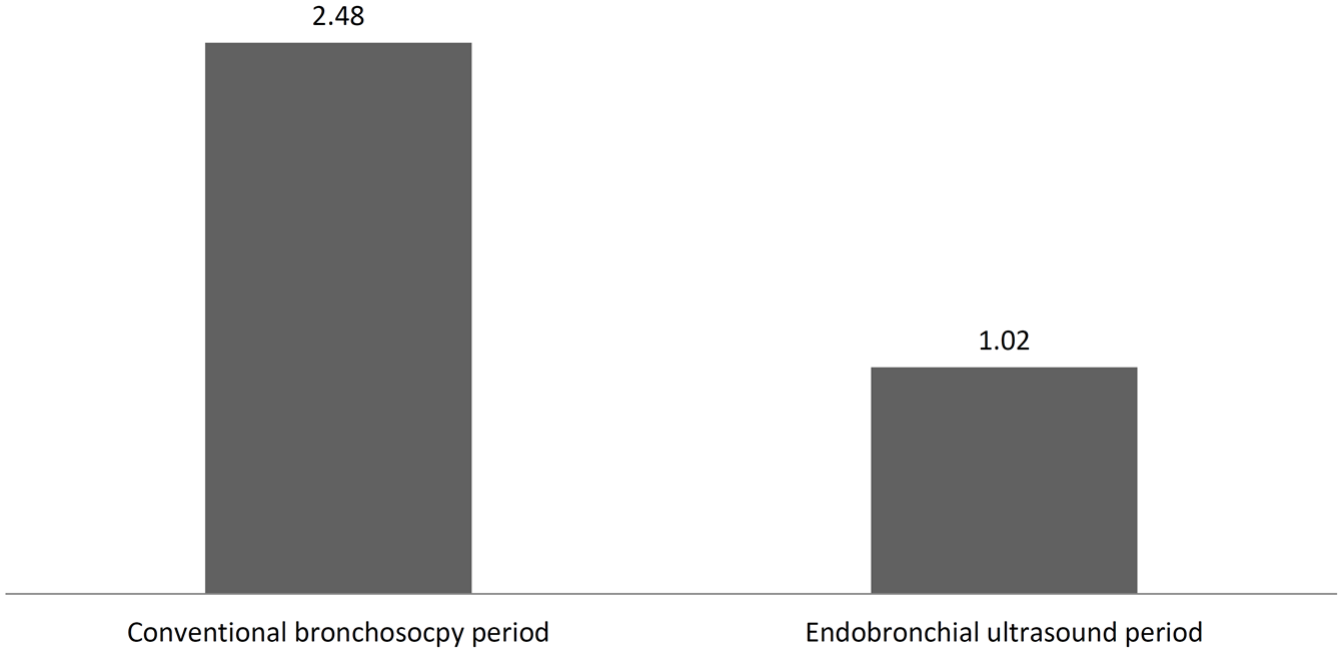
The incidence (%) of iatrogenic pneumothorax after bronchoscopy.

**Fig 3:**
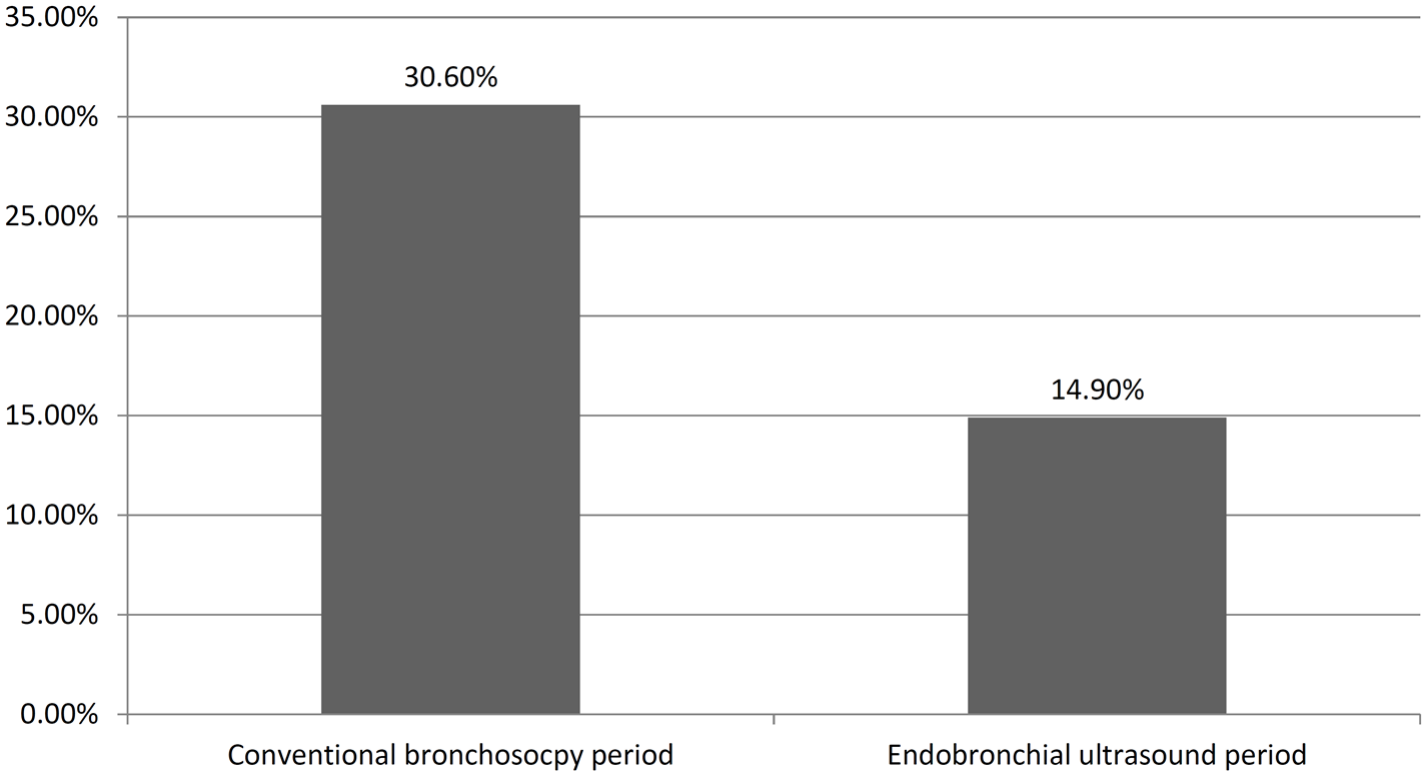
The proportion of patients who needed a CT-guided biopsy for diagnosis after failure of bronchoscopy.

**Fig 4:**
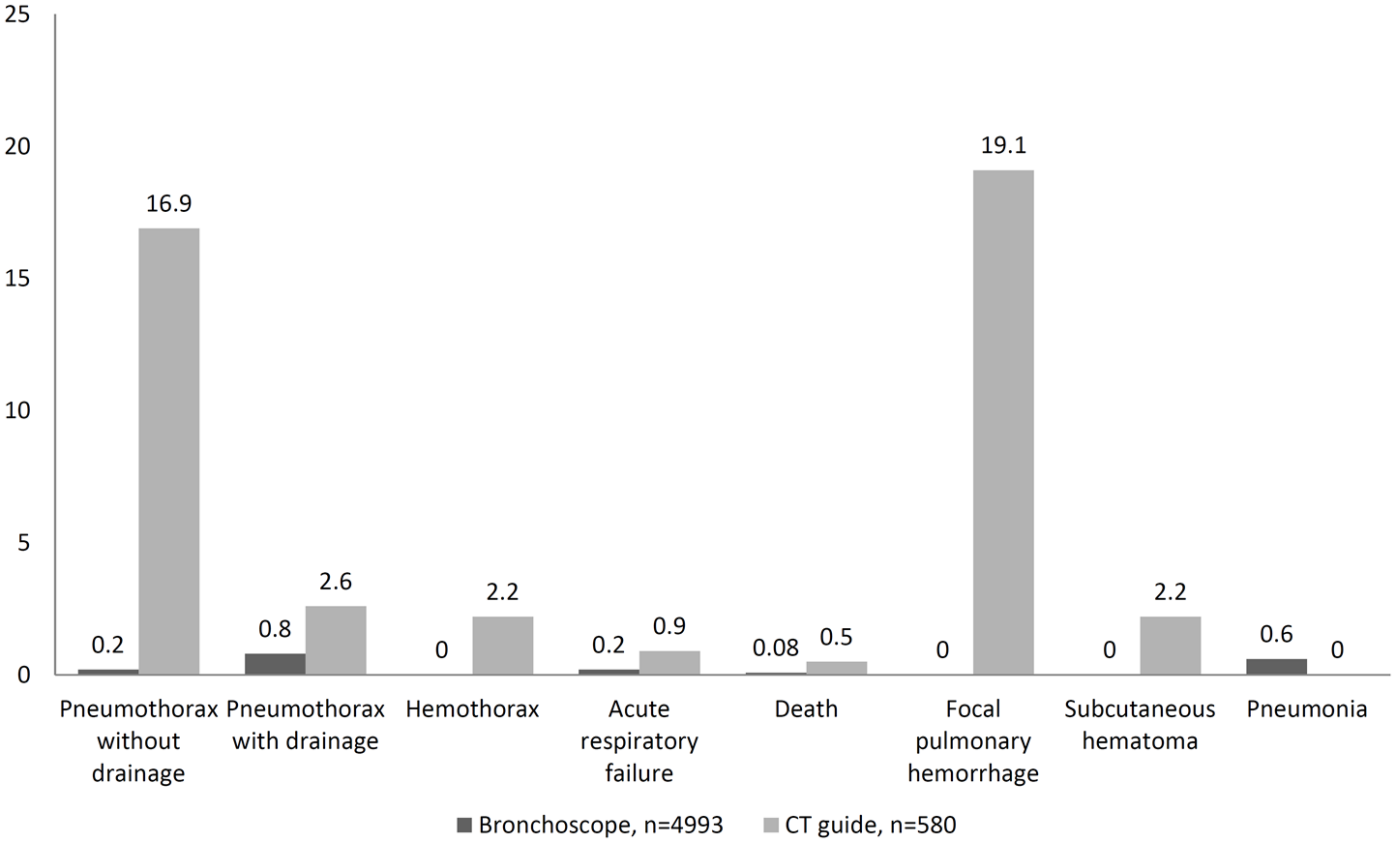
The comparative complication rates of bronchoscopy and CT-guided biopsy during the endobronchial bronchoscopy period.

The incidence of iatrogenic pneumothorax after bronchoscopy decreased after the introduction of EBUS (2.48% in conventional bronchoscopy compared with 1.02% in EBUS, P = 0.017, Fig 2). Moreover, the proportion of patients who required a CT-guided biopsy for diagnosis after failure of bronchoscopy significantly decreased by 15.70% after the introduction of EBUS (P < 0.0001, Fig 3). In the EBUS period, there were 4993 and 580 patients who underwent bronchoscopic and CT-guide biopsy for pulmonary lesions, respectively. Comparing the diagnostic complications arising from CT-guided biopsy and bronchoscopy in the EBUS period, the incidence of pneumothorax (19.7% vs. 1%, P < 0.0001), hemothorax (2.2% vs. 0%, P < 0.0001), pulmonary hemorrhage (19.1% vs. 0%, P < 0.0001), subcutaneous hematoma (2.2% vs. 0%, P < 0.0001), acute respiratory failure (0.9% vs. 0.2%, P = 0.015), and death (0.5% vs. 0.08%, P = 0.028) were lower in the EBUS group. However, the occurrence rate of iatrogenic pneumonia was slightly higher for bronchoscopy (0% vs. 0.6%, P = 0.068, Fig 4).

## Discussion

Our study demonstrates that lung cancer diagnostic methods have significantly changed after the introduction of R-EBUS and EBUS-TBNA. Comparing the conventional bronchoscopy and EBUS periods, the number of lung cancer cases diagnosed by bronchoscopy significantly increased. There has also been an increase in the proportion of patients proceeding directly to surgery without pathological confirmation of lung cancer diagnosis. Moreover, there has been a significant reduction in CT-guided biopsy and pleural effusion cytology.

### Diagnostic modalities

The American College of Chest Physicians (ACCP) observed that the overall diagnostic sensitivity of conventional bronchoscopy in lung cancer was 88% (range: 67–97%) and 78% (range: 36–88%) for central and peripheral carcinoma, respectively. The sensitivity of conventional bronchoscopy is high for the detection of endobronchial disease and poor for peripheral lesions <2 cm in diameter[11]. The diagnostic yield of conventional bronchoscopy was 63% for lesions ≥ 2 cm (n = 984), and 34% for lesions < 2 cm (n = 383)[12]. Endobronchial ultrasound has broadened the diagnostic potential of bronchoscopy. It exists in two forms: radial and linear probe EBUS. Radial probe endobronchial ultrasound has improved the rate of peripheral lung cancer diagnosis[13]. One study reported a point specificity of 1.00 (95% confidence interval [CI]: 0.99–1.00) and a point sensitivity of 0.73 (95% CI: 0.70–0.76) for the detection of lung cancer [14]. When a concentric radial ultrasound image was obtained, the diagnostic yield of bronchoscopy was 84%, which is comparable to that of CT-TNAB [2]. Furthermore, EBUS-TBNA has played a key role in the diagnosis of mediastinal, near centrally located lesions without endobronchial involvement, as well as in lymph node staging for lung cancer[15–18]. It has a high sensitivity of 90%, specificity of 100%, negative predictive value of 83%, positive predictive value of 100%, and diagnostic accuracy of 93%[19]. It is also a minimally invasive method of mediastinal biopsy but achieves similar results for the mediastinal staging of lung cancer[20]. Before the introduction of EBUS, CT-guided biopsy was the alternative diagnostic choice if conventional bronchoscopy returned a negative result. With the widespread use of the R-EBUS and EBUS-TBNA diagnostic methods in lung cancer diagnosis, the use of CT-guided biopsy significantly decreased as either an initial or an alternative choice (Figs 1 and 3).

Interestingly, more patients are being referred directly to surgery without pre-operative confirmation of the histological diagnosis, as is evident from the increase in histological diagnoses made from surgery from 15% in the conventional bronchoscopy period to 20.7% in the EBUS period. The reason may be that the National Lung Screening Trial (NLST), in comparison with the radiography group, found that screening with the use of low-dose CT (LDCT) in high risk patients reduces mortality from lung cancer[21]. With the widespread use of LDCT, the frequency of discovering ground glass opacity (GGO) pulmonary lesions has increased. Additionally, R-EBUS is a useful tool for precise localization of peripheral solid pulmonary lesions, but there have been no detailed reports about the use of R-EBUS images for GGO. In a recent limited report, transbronchial biopsy (TBB) through EBUS with a guide sheath (EBUS-GS) can be considered as one of the diagnostic methods for GGO, and the overall diagnostic yield of EBUS-GS TBB was 65.0% (26 of 40 lesions). However, further technological development is required to identify the location of the target GGO lesion more precisely[22]. The number of lung cancer patients by surgical diagnosis directly increased due to the lack of experience with R-EBUS images for GGO in our institution.

### Lung cancer histological subtypes

Adenocarcinoma presented as a peripheral mass in 61% of cases and in 38.3% as a central lesion. Squamous cell carcinoma presented as a central lesion (72.2%) more commonly than as a peripheral lesion (27.8%)[23]. Adenocarcinoma in situ (AIS) is categorized as a preinvasive lesion for lung adenocarcinoma, and refers to a small nodule with purely lepidic (bronchioloalveolar) growth without stromal, vascular, or pleural invasion. On CT, AIS appears as a pure small GGO and is typically larger than 5 mm[24]. Pulmonary metastases typically appear as peripheral, rounded nodules of variable size, scattered throughout both lungs[25]. Our study showed that lung cancer histological subtypes had significantly changed between these two periods. After the introduction of EBUS and widespread use of LDCT for lung cancer screening, lung cancers diagnosed as adenocarcinoma and AIS increased. One reason for this increase may be the correlation with the increasing use of EBUS and surgery as diagnostic modalities for PPLs. The other reason is that the incidence of lung adenocarcinoma in women has risen over the last 30 years. This differs from the trend for squamous cell lung cancer in women and both subtypes in men, which have all been decreasing in recent years[26]. In our study, the number of squamous carcinoma patients factually decreased in the EBUS period.

The rate of non-small-cell lung carcinoma not otherwise specified (NSCLC-NOS) was reduced in patients who underwent immunohistochemistry[27]. This reduction in the diagnosis of NSCLC-NOS from 5.0% in the conventional bronchoscopy period to 1.5% in the EBUS period was also seen in this study.

### Complications

Pneumothorax is the most common complication of needle aspiration or biopsy of the lung, and is reported to occur in 17–26.6% of patients. The chest tube insertion rate is much lower, ranging from 1% to 14.2% of patients[28,29]. Pulmonary hemorrhage is the second most common complication of needle biopsy of the chest, with reported frequencies ranging from 4% to 27% [25]. Previous meta-analyses of conventional TBNA showed a major complication rate of 0.3%. Massive hemorrhage, pneumomediastinum, pneumothorax requiring drainage, cardiac tamponade, and hemomediastinum have all been reported as TBNA complications[30]. A meta-analysis of EBUS-TBNA showed a complication rate of 0.15% (2 of 1299 cases), with only one case showing pneumothorax requiring drainage as a major complication [31]. Another study in 2010 showed a complication rate of 0.46% for EBUS-TBNA[32]. One study previously compared the two methods head-to-head, and overall complication rates were higher (27% vs. 3%, P = 0.03) in those undergoing CT-guided percutaneous needle biopsy (CT-PNB), while the diagnostic accuracy of EBUS-TBB was not inferior to CT-PNB[33]. Previous studies reported similar results. In the EBUS period, comparing complication rates between CT-guided biopsy and diagnostic bronchoscopy, including pneumothorax, hemothorax, acute respiratory failure, pulmonary hemorrhage, subcutaneous hematoma, and death, revealed that the occurrence rates were all lower for bronchoscopy (Fig 4). In addition to clinical performance, the optimal method for diagnosing PPLs may also be influenced by the costs of the individual procedures. One study indicated that the costs of EBUS-TBB and CT-PNB to evaluate PPL appear to be equivalent, but specific clinical-radiologic factors known to influence procedural outcomes will influence cost-benefit outcomes[34]. This study demonstrates that the introduction of R-EBUS and EBUS-TBNA has resulted in significant changes to the clinical diagnostic modalities of lung cancer and reduced the incidence of complications after the diagnostic procedure. In the future, advanced bronchoscopic techniques may be widely used in the diagnostic workup of lung cancer.

### Limitations

Our study has some limitations. First, it was a retrospective observational study, which means that only patients who were clinically selected for each diagnostic modality were included. Blinded, randomized, and controlled trials are hard to perform in these subjects due to the practices. Second, these data were from an institution with expertise in R-EBUS and EBUS-TBNA technics. It may not be the same in other institutions. Compared to other modalities, we think EBUS diagnostic methods will play important role in the future, and may become incorporated in a training program in the respiratory medicine and cardiothoracic surgery specialties.

## Conclusion

At our institution, the increasing use of R-EBUS and EBUS-TBNA for providing lung cancer diagnosis has led to a significant reduction in other diagnostic modalities, such as CT-guided biopsy and pleural effusion cytology. These changes in practice also led to a reduction in the incidence of complications. Thus, it is not only an excellent diagnostic method but also minimizes the risk to the patient. In addition to diagnostic accuracy, minimally invasive intervention and patient safety are the major concerns of clinical physicians.

## Abbreviations

EBUS: Endobronchial ultrasound
R-EBUS: Radial probe endobronchial ultrasound
EBUS-TBNA: Endobronchial ultrasound-guided transbronchial needle aspiration
CT: Computed tomography
PLLs: Peripheral lung lesions
CT-PNB: Computed tomography-guided percutaneous needle biopsy
GGO: Ground glass opacity
TBB: Transbronchial biopsy
